# Probiotics Reduce Health Care Cost and Societal Impact of Flu-Like Respiratory Tract Infections in the USA: An Economic Modeling Study

**DOI:** 10.3389/fphar.2019.00980

**Published:** 2019-08-28

**Authors:** Irene Lenoir-Wijnkoop, Dan Merenstein, Daria Korchagina, Christa Broholm, Mary Ellen Sanders, Dan Tancredi

**Affiliations:** ^1^Department of Pharmaceutical Sciences, University of Utrecht, Utrecht, Netherlands; ^2^Family Medicine Department, Georgetown University, Washington, DC, United States; ^3^Real World Insights, IQVIA, Paris, France; ^4^Chr. Hansen A/S, Human Health Innovation, Hoersholm, Denmark; ^5^Dairy & Food Culture Technologies, Centennial, CO, United States; ^6^Department of Pediatrics and the Center for Healthcare Policy and Research, University of California, Davis, Sacramento, CA, United States

**Keywords:** probiotics, health economics, respiratory tract infection, influenza, cost savings

## Abstract

Acute respiratory tract infections (RTIs) of viral origin place a substantial burden on health care resources and society. Randomized controlled trials have shown positive effects of probiotics on clinical outcomes in these commonly occurring RTIs. Two meta-analyses published by the York Health Economics Consortium (YHEC) and Cochrane reported the efficacy of probiotics in reducing incidence and duration of RTIs, number of antibiotic courses, and days absent from work. The aim of this study was to assess the potential health-economic impact of probiotics on RTI-associated events and expenses in the US primary care setting. A state-transition microsimulation model reproduced a study population representative of the US national demographics for age and gender (1/1,000 sample). RTI incidence was based on the influenza-like illness outpatient consultation rate reported by the Centers for Disease Control and Prevention (CDC) FluView. Data on vaccination, on factors that negatively impact RTI outcomes, on resource utilization, and on productivity loss were obtained from US national databases. Analyses were performed for both meta-analyses independently. Outcomes included cost savings for the health care payer, related to a reduced number of RTI episodes, less outpatient consultations, and decreased medical prescriptions as well as cost savings from a broader societal perspective related to productivity loss. The analysis showed that generalized probiotic intake in the US population for 2017–2018 would have allowed cost savings for the health care payer of 4.6 million USD based on the YHEC scenario and 373 million USD for the Cochrane scenario, by averting 19 million and 54.5 million RTI sick days, respectively, compared to no probiotics. Antibiotic prescriptions decreased with 1.39–2.16 million courses, whereas absence from work decreased by 3.58–4.2 million days when applying the YHEC and Cochrane data, respectively. When productivity loss is included, total savings for society represented 784 million or 1.4 billion USD for the YHEC and Cochrane scenarios, respectively. Subgroup analyses demonstrated an incremental benefit of probiotics in at-risk groups, which might be of relevance for targeted interventions. Sensitivity analyses confirmed the robustness of the model outcomes. Our analysis demonstrated a positive impact of probiotics on the health care and economic burden of flu-like RTIs. Improved disease outcomes translated into considerable cost savings for both the payer and society.

## Introduction

Acute respiratory tract infection (RTI) is a frequent illness, generally of viral origin. Clinical conditions range from mild cold symptoms to influenza, the most serious form of common acute RTI. In most health care settings, diagnostic tests that would differentiate between influenza and other forms of viral RTIs are not routinely performed. Although most acute RTI episodes resolve spontaneously (Dasaraju and Liu, 1996), RTIs result in a high number of outpatient consultations and pose a heavy burden on society and health care systems. Strategies to reduce the incidence and effects of common acute RTIs attract major public health interest, given the large number of individuals affected each year as well as the impact on patient health outcomes and on medical and personal costs. In order to facilitate disease monitoring, this overlapping group of acute viral respiratory infections is generally referred to as influenza-like illness (ILI) (Fowlkes et al., 2014). Recently, the WHO defined ILI as “an acute respiratory illness with a measured temperature of >38°C and cough, with [symptom] onset within the past 10 days” (Fitzner et al., 2018). In the USA, information on outpatient visits to health care providers for ILI is collected through the US Outpatient Influenza-like Illness Surveillance Network (CDC ILInet, 2018). For this system, ILI is defined as having a fever (>100°F or >37.8°C) and cough and/or sore throat (in the absence of a known cause).

Probiotics are live microorganisms that, when administered in adequate amounts, confer a health benefit on the host (Hill et al., 2014). Interest in the potential impact of probiotics on health outcomes has been increasing in recent years. This impact has been investigated in several therapeutic areas, including RTIs. According to a recent survey among health care providers who routinely prescribe medication, 61% had recommended probiotic food or supplements to their patients (Draper et al., 2017). Several clinical studies have evaluated the effectiveness of probiotics when administered to healthy subjects in reducing the incidence and duration of infectious respiratory conditions (de Vrese et al., 2005; Leyer et al., 2009; Berggren et al., 2011). Two large meta-analyses have investigated the preventative effect of taking probiotics versus placebo. The York Health Economics Consortium (YHEC) performed a systematic review and meta-analysis on the duration of illness in healthy children and adults who developed acute respiratory infectious conditions (King et al., 2014); results showed that probiotics significantly reduced RTI episode duration. The Cochrane Collaboration assessed the effectiveness of probiotics, compared with placebo, in the prevention of acute upper RTIs in healthy people of all ages and reported that probiotics reduced RTI incidence and antibiotic prescription rate (Hao et al., 2015).

## Objectives

Based on the above-mentioned meta-analyses reporting the positive outcomes of probiotics in RTI, we hypothesized that generalized use of probiotics would meaningfully reduce RTI duration and/or frequency and thus the use of health care resources and related expenses for RTI in the USA. The objective of this study was to quantify the effect of probiotics on RTI-related health and cost outcomes in the US primary care setting. The analysis also explored the effect of probiotic intake on productivity loss.

## Methods

### Model Description

**Figure 1:**
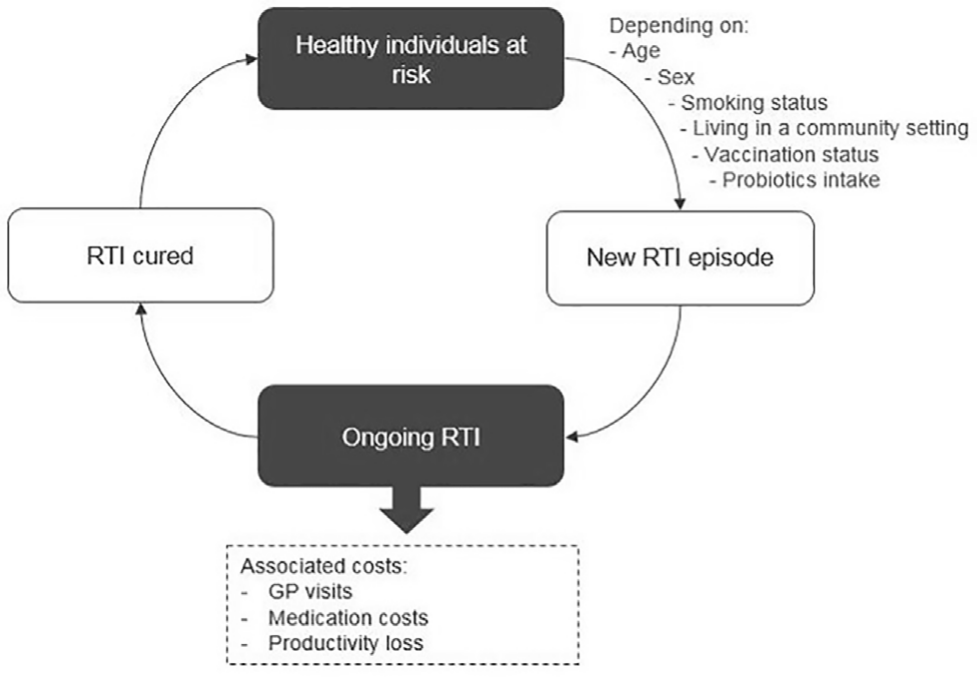
Model structure.

Our economic analysis compared generalized probiotic intake versus no probiotic intake. We used a state-transition microsimulation model, which enabled us to track the disease pathway of each subject, accumulating costs and events dependent on individual baseline and/or time-varying characteristics. Two previously published economic evaluations of probiotics in RTIs inspired the model structure (Lenoir-Wijnkoop et al., 2015; Lenoir-Wijnkoop et al., 2016). The study cohort was a representative sample of the US population in terms of demographics and known RTI-related risk factors. Model convergence was tested in order to ensure that the number of individuals in the analysis was sufficient to obtain robust results. The model comprised two health states, “at risk” and “ongoing RTI” (**Figure 1**).

All individuals in the cohort were evaluated under each of the two probiotic intake regimens, generalized probiotic use versus no probiotic use. For the analysis of each regimen, each subject started in the “at-risk” state and could move to “ongoing RTI” according to predefined transition probabilities, calculated based on US epidemiology data. The cycle length was 1 day, and the time horizon was 1 year, reflecting the 2017–2018 influenza season of FluView data from the Centers for Disease Control and Prevention (CDC).

### Model Inputs and Data Sources

#### Probiotic Effect

The clinical effects of probiotics were obtained from the meta-analyses published by the YHEC (King et al., 2014) and Cochrane (Hao et al., 2015). These were used to conduct two independent scenario analyses comparing generalized probiotic use versus non-use, each based on different assumptions: YHEC showed a significantly shorter duration of −0.77 days [−1.50 to −0.04] on an average duration of 7.4 days per episode of RTI, among otherwise healthy children and adults taking probiotics compared to those taking placebo. The Cochrane study reported that probiotics significantly reduced RTI duration by 1.89 days [1.75 to 2.03] per episode of an average duration of 8.82 days and RTI incidence by 30% (RR = 0.70 [0.50 to 0.84]) (**Table 1**). The authors also found a significant reduction of the antibiotic prescription rate of 35% (RR = 0.65 [0.45 to 0.94]), which was applied to both scenarios. Additionally, the YHEC meta-analysis studied the impact of probiotics on work absenteeism. The reported standardized mean difference (SMD) in the number of days absent from work was used to estimate the impact of probiotics on productivity loss. The Cochrane meta-analysis focused on unvaccinated individuals; therefore, no impact of probiotics sourced from the Cochrane meta-analysis was applied in vaccinated patients.

The clinical effects of probiotics were obtained from the meta-analyses published by the YHEC (King et al., 2014) and Cochrane (Hao et al., 2015). These were used to conduct two independent scenario analyses comparing generalized probiotic use versus non-use, each based on different assumptions: YHEC showed a significantly shorter duration of −0.77 days [−1.50 to −0.04] on an average duration of 7.4 days per episode of RTI, among otherwise healthy children and adults taking probiotics compared to those taking placebo. The Cochrane study reported that probiotics significantly reduced RTI duration by 1.89 days [1.75 to 2.03] per episode of an average duration of 8.82 days and RTI incidence of 0.70 [0.50 to 0.84] (**Table 1**). The authors also found a significant reduction of the antibiotic prescription rate of 0.65 [0.45 to 0.94], which was applied to both scenarios. Additionally, the YHEC meta-analysis studied the impact of probiotics on work absenteeism. The reported standardized mean difference (SMD) in the number of days absent from work was used to estimate the impact of probiotics on productivity loss. The Cochrane meta-analysis focused on unvaccinated individuals; therefore, no impact of probiotics sourced from the Cochrane meta-analysis was applied in vaccinated patients.

**Table 1:** Summary of model inputs—epidemiological and resource utilization parameters.

Model parameters				Reference
Influenza vaccination coverage				CDC Fluvaxview, 2018
**Steps to ILI incidence estimation**				
All cause consultations, all ages				NAMCS
Total ILI consultations, all ages				CDC FluView, 2018
**Clinical effect of probiotics**				
YHEC	On RTI incidence: NA/On RTI duration: –0.77 days vs placebo/On antibiotic use: NA/On work absenteeism: –0.17 SMD			King et al., 2014
Cochrane	On RTI incidence: RR = 0.70* vs placebo/On RTI duration: –1.89 days vs placebo/On antibiotic use: RR = 0.65 vs placebo/On work absenteeism: NA			Hao et al., 2015
**Risk factors**				
*Smoking*				
Active smokers				CDC MMWR, 2018a, CDC MMWR, 2018b
Passive smokers				National Cancer Institute, 2017
*Shared indoor environment*				
School enrollment				United States Census Bureau, 2018b
Employment status				Bureau of Labor Statistics, 2018
Living in a nursing home				Henry J Kaiser Family Foundation, 2019b
**Impact of risk factors on RTI**				
*Smoking*				
Active smokers	On RTI incidence: NA/On RTI duration: +16.8% vs no smokers			Benseñor et al., 2001
Passive smokers	On RTI incidence: RR = 1.15/On RTI duration: +4.5% vs no smokers			Benseñor et al., 2001
*Shared indoor environment*				
Day care (including school) vs home care	On RTI incidence: RR = 1.22/On RTI incidence: NA			Louhiala et al., 1995
Shared office vs alone	On RTI incidence: RR = 1.07/On RTI incidence: NA			Jaakkola and Heinonen, 1995
**Cost parameters**				
*Direct cost parameters*	**% using the resource**	**Unit cost (by payer) (USD)**	**Copayment (USD)**	
PCP cost^#^	100%	74.16**	25**	Physician Fee Schedule
Antibiotics (amoxicillin)^§^	29%	6.49	0	Medi-Span Price Rx 2018
Non-antibiotic medication	56.62%	26.59	11	Karve et al., 2013
*Indirect cost parameters*	**% missed days**	**Number of missed days, mean (SD)**	**Cost per day lost (employer) (USD)** **^†^**	
Employee with ILI	42%	1.7 (5.1)	217.92	Palmer et al., 2010, Bureau of Labor Statistics, 2018
Sick children with ILI	18%	0.5 (1.5)	217.92	Palmer et al., 2010, Bureau of Labor Statistics, 2018

SMD, standardized mean difference; NA, not applicable; CDC, Centers for Disease Control and Prevention; ILI, influenza-like illness; NAMCS, National Ambulatory Medical Care Survey; YHEC, York Health Economics Consortium; RTI, respiratory tract infection; RR, risk ratio; MMWR, Morbidity and Mortality Weekly Report; PCP, primary care physician.

*Transformed from OR to RR using exact numbers and sample size.

**Reimbursed unit price of current procedural terminology code 99213.

^#^Published by the Centers for Medicare & Medicaid Services.

^§^Commonly used and recommended for by the CDC.

^†^Cost per absent day is based on daily wage from (Bureau of Labor Statistics, 2018).

#### Demographic Structure of the Study Population

The demographic data by age and gender were obtained from the United States Census Bureau (2018a).

#### Respiratory Tract Infection Incidence and Vaccination Status

The daily RTI incidence probabilities were estimated based on the ILI outpatient consultation rate as reported by CDC FluView (2018). The vaccination status of American citizens was taken into account in the base case to allow exclusion of a probiotic effect in vaccinated patients, as the Cochrane scenario excluded vaccinated subjects. That is, for a vaccinated patient, we specified that RTI incidence and duration were identical between the probiotic and no-probiotic scenarios, effectively excluding probiotic effects for vaccinated patients. The prevalence of influenza vaccination in the USA was obtained from the CDC Fluvaxview (2018). The lower probability of getting ILI for vaccinated subjects was estimated based on the vaccination effect reported by two meta-analyses, Jefferson et al., (2008) and Demicheli et al. (2018) for children and adults, respectively.

#### Risk Factors

Several risk factors other than age are known to have an impact on RTI, such as smoking or a daily shared indoor environment. To guarantee stable and robust results, these risk factors were not part of the base case but were included in subgroup analyses that assessed the variability of the results across different subpopulations and identified subpopulations likely to benefit more from the use of probiotics.

Benseñor et al. (2001) carried out a randomized controlled trial (RCT) among 39,876 female participants to assess active and passive smoking in relation to frequency of colds. The study showed no significant impact of active smoking on upper RTI incidence, while it significantly increased the risk of having a longer duration (RR > 7 days) of 1.62 [1.40 to 1.87] for light smokers and 2.63 [2.02 to 3.44] for heavy smokers. In passive smokers, a higher RTI incidence [1.15 (1.05 to 1.26)] and a longer RTI duration per episode [4.5 (0.1 to 8.9)] were observed in comparison to non-smokers.

The CDC Morbidity and Mortality Weekly Report (MMWR) published the prevalence of active smoking among adults (CDC MMWR, 2018a) as well as among middle and high school students (CDC MMWR, 2018b). The prevalence of passive smoking was obtained from the National Cancer Institute (2017).

Daily shared indoor environments (i.e. children studying in school and adults working in a shared office) have been shown to increase the risk of acquiring an RTI, with an associated impact on RTI incidence (Dasaraju and Liu, 1996). Children in day care centers, aged 1–7 years, appeared to have a significantly higher risk of getting a respiratory infection than children staying at home [1.22 (1.13 to 1.31)] (Louhiala et al., 1995). In the model, this effect was also applied for children aged 8–15 years. The results of a study on adults working in a shared office environment showed a higher risk of having more than two cold episodes during a 12-month period [1.64 (1.08 to 2.50)] (Jaakkola and Heinonen, 1995). Prevalence of shared indoor environments was based on school enrollment (United States Census Bureau, 2017), employment status (Bureau of Labor Statistics, 2018), and proportion of people living in a nursing home (Henry J Kaiser Family Foundation, 2019b).

#### Respiratory Tract Infection-Related Costs

Resource utilization consisted of estimated consultation fees for a primary care physician (PCP), cost of antibiotics and other prescribed medication resource use, and copayment (Henry J Kaiser Family Foundation, 2017a). Inpatient costs were not considered. Cost for consulting a PCP was taken from the Physician Fee Schedule (Centers for Medicare & Medicaid Services, 2018) and based on the assumption of a single PCP consultation per RTI episode to align with the estimates of the RTI incidence derived from the number of ILI outpatient visits, collected through the US Outpatient Influenza-like Illness Surveillance Network (CDC ILInet, 2018). The cost of antibiotics was based on a recent study that reported an antibiotic prescription during influenza seasons of 29% (Havers et al., 2018). Due to the lack of data availability, prescribed medications other than antibiotics were included in this analysis as a single cost item, based on a publication that assessed health care costs associated with influenza (Karve et al., 2013). The cost of over-the-counter medication among the general population was not included, as reliable information on cost estimates was insufficient.

A broader societal perspective was taken by combining the cost covered by insurance/copayment and ILI-related productivity losses (Palmer et al., 2010). An overview of all data inputs and sources is presented in **Table 1**.

#### Statistical Analyses

The model was used to quantify the impact of generalized probiotic use versus no probiotic use on each of the following outcomes: number of ILI events, number of ILI days, number of antibiotic prescriptions, number of days missed from work, PCP visit costs, medication costs, and productivity loss. Subgroup analyses were conducted on age, vaccination status, smoking status (active and passive), and living or working in a shared indoor environment. Two additional scenario analyses were conducted: (1) comparing a population with generalized probiotic intake versus a population with current probiotic intake in the USA and (2) considering an alternative data source for productivity loss in ILI patients.

Uncertainty around model results due to model assumptions was further explored in a one-way sensitivity analysis, which considered two key parameters: avoided RTI days and saved total societal cost with probiotic use versus no probiotic use (**Table 2**). All statistical analyses were performed and produced in Microsoft Excel (2016), and the model was developed with the utilization of Visual Basic for Applications (VBA) in Excel.

**Table 2:** Sensitivity analyses: lower and upper bounds of variation for model parameters.

Parameter	Value			Source
	Base case	Lower	Upper	
**YHEC scenario (probiotic effects)**				
Change duration per RTI episode	0.77	0.04	1.5	95% CI, King et al., 2014
Reduced antibiotic prescription (RR)*	0.65	0.45	0.94	95% CI, Hao et al., 2015
Change in loss of productivity, adults	0.87	0.153	1.581	95% CI, King et al., 2014 + assumption
Change in loss of productivity, children^‡^	0.26	0.045	0.465	
**Cochrane scenario (probiotic effects)**				
Change in duration per RTI* episode	1.89	1.75	2.03	95% CI, Hao et al., 2015
Reduced incidence of RTI* (RR)	0.70	0.5	0.84	
Reduced antibiotic prescription* (RR)	0.65	0.45	0.94	
Change in loss of productivity, adults	0.87	0.153	1.581	95% CI, King et al., 2014 + assumption
Change in loss of productivity, children^‡^	0.26	0.045	0.465	
**Both scenarios**				
Probability of non-antibiotic medication	56.62%	50.00%	60.00%	Assumption based on expert opinion
Antibiotic cost, 0–14 years	2.95	1.48	4.43	±50% of base case value
Antibiotic cost, 15+ years	3.54	1.77	5.31	
PCP cost	99.16	69.64	124.44	Codes 99212 and 99214, Physician Fee Schedule

Upper and lower limits represent 95% confidence interval as reported by the indicated source. PCP, primary care physician; RR, risk ratio; RTI, respiratory tract infection; YHEC, York Health Economics Consortium; PCP, primary care physician.

*Applied in non-vaccinated individuals only.

^‡^Productivity loss caused by sick child.

### Model Validity

**Figure 2:**
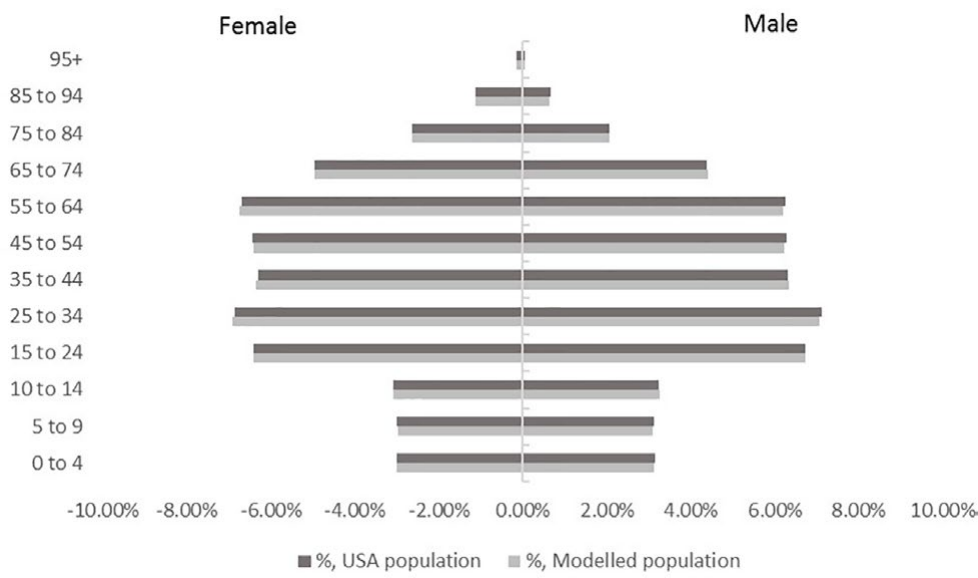
Population structure by age and gender in the model cohort versus USA population.

A cohort of 329,256 individuals was generated based on the chosen sample rate. The model demonstrated a high precision in simulating the US population structure by age and gender (**Figure 2**). High accuracy was also achieved in simulating risk factor prevalence as well as ILI event incidence with <0.2% difference in total number of RTI events when compared to the FluView data.

To ensure that the model provides robust results with a chosen sample size, convergence testing was conducted and confirmed the stability of estimates for both the YHEC and the Cochrane scenarios.

## Results

In the YHEC scenario, the base case analysis showed that the shorter duration of disease associated with probiotic intake engendered 19,012 fewer acute RTI days compared to no probiotics, while 1,393 antibiotic courses (−19.4%) were avoided in the evaluated study cohort (**Table 3**). As the YHEC meta-analysis did not investigate the effect of probiotics on RTI incidence, the difference in the number of RTI episodes and related physician consultations was not part of the base case analysis. When these outcomes are projected to the actual US population, this translates to potential cost savings associated with probiotic use of 4.6 million USD for the health care payer. When productivity loss related to absence from work is included, probiotic intake has the potential for additional total cost savings of 784 million USD for the USA.

**Table 3:** York Health Economics Consortium (YHEC) scenario: impact of probiotics on RTI-related events versus no probiotics (sample size 1/1,000).

Event outcome	Probiotics	No probiotics	Difference	Difference in %
RTI days	163,701	182,713	−19,012	−10.41%
No. of antibiotic courses	5,804	7,197	−1,393	−19.36%
No. of missed work days	3,397	6,973	−3,576	−51.29%

RTI, respiratory tract infection.

In the Cochrane scenario, the base case analysis of the effect of probiotics on both a reduced RTI incidence and a shorter disease duration demonstrated a decrease of 4,103 RTI episodes, a reduction of 54,491 RTI days, and 2,166 antibiotics courses averted in the probiotic arm compared to the arm without probiotics (**Table 4**). Projection of the base case outcomes to the US population showed that probiotic use would result in cost savings of 373 million USD for the health care payer. For this scenario, when generalized probiotic use versus no probiotic use are compared, the total decrease in RTI-associated expenses due to medical resource utilization and productivity loss combined equaled 1.4 billion USD.

**Table 4:** Cochrane scenario: impact of probiotics on RTI-related events versus no probiotics (sample size 1/1,000).

Event outcome	Probiotics	No probiotics	Difference	Difference in %
RTI episodes	20,568	24,671	−4,103	−16.63%
RTI days	163,107	217,598	−54,491	−25.04%
No. of antibiotic courses	5,026	7,192	−2,166	−30.12%
No. of missed work days	2,753	6,971	−4,217	−60.50%

RTI, respiratory tract infection.

Subgroup analyses of risk factors showed that an incremental benefit of probiotics was observed in children aged ≤15 years, in individuals sharing a daily indoor environment, and in passive smokers. The positive impact of probiotics was highest in the pediatric population that constituted 19.8% of the cohort population, but in which probiotic use contributed to 41.3% of avoided RTI days and 26.3% of the total cost savings. Results of the unvaccinated subgroup analysis showed that the 53.7% of unvaccinated individuals in the model population contributed to 56% of avoided RTI days and 63.9% of saved total costs. The outcomes of the subgroup analyses are summarized in **Table 5**.

**Table 5:** Subgroup analysis by risk factors (age, smoking, and living in a shared daily environment) (sample size 1/1,000).

Subgroup	% of model population	% of avoided RTI days	% of total societal cost savings
YHEC scenario			
Children (aged 0–15)	19.81%	41.30%	26.29%*
Passive smokers	26.59%	34.60%	30.03%
Individuals with shared indoor environments	53.19%	55.55%	71.88%
Unvaccinated individuals	53.70%	56.10%	63.87%
**Cochrane scenario**			
Children (aged 0–15)	19.81%	34.35%	34.62%*
Active smokers	12.50%	11.93%	13.13%
Passive smokers	26.59%	34.11%	31.23%
Individuals with shared indoor environments	53.19%	58.25%	67.24%

RTI, respiratory tract infection; YHEC, York Health Economics Consortium.

*Excluding productivity loss.

In the base case analysis, current probiotic intake was disregarded even though probiotic consumption would be expected to influence the reported RTI incidences and subsequently our study outcomes. Therefore, a scenario analysis was carried out based on estimated probiotic intake. According to the National Health Interview Survey (NHIS), 1.6% of American adults take probiotics and/or prebiotics as dietary supplements (Clarke et al., 2015). Further, it has been estimated that dietary supplements account for about 36% of the probiotic sales in the USA; the remaining 64% of the market represents thus probiotic foods (Vanderhoof and Young, 2008). Therefore, an overall 4.4% probiotic intake among the US population was used to include current probiotic consumption in a scenario analysis. This showed that outcomes were only slightly different from the base case, confirming the robustness of the model.

## Discussion

We developed a state-transition microsimulation model to quantify the effect of probiotics on RTI-related health and cost outcomes in the USA. The analysis considered the impact of generalized probiotic intake from a health care payer’s perspective in primary care, as well as in a broader societal setting by including productivity loss associated with flu-like RTI. The model accurately reproduced the US population structure, the incidence pattern, and the risk factor prevalence over the study period. Two meta-analyses reported clinical benefits of probiotics, one showing a reduction of disease duration and the other both a decrease of disease incidence and duration (**Table 1**). While the changes are small at an individual level, when applied to the US population, the health impact is sizable, ranging from an estimated 19,012,000 to 54,491,000 fewer RTI days, 1,393,000 to 2,166,000 averted antibiotic courses, and 3,576,000 to 4,217,000 avoided missed work days, according to the YHEC scenario and the Cochrane scenario, respectively. The Cochrane scenario showed a higher overall impact on public health, due to the fact that it considered both a shorter RTI duration and a reduced RTI incidence. In terms of cost savings, the impact of avoided RTI events, translated to the US population, represented a potential total amount of approximately 1.4 billion USD, of which 370 million USD represents savings for the health care payer.

When cross-validation is performed, the findings of this US analysis were consistent with the previously published French and Canadian assessments (Lenoir-Wijnkoop et al., 2015; Lenoir-Wijnkoop et al., 2016). The proportion of avoided RTI days in the population with probiotics versus no probiotics in the models was similar to the results in the current analysis for both the YHEC and Cochrane scenarios. Differences in other outcomes were to be expected due to country-specific characteristics, related to vaccination coverage, prescription patterns, cost inputs, or different conditions in absence from work.

### Strengths and Limitations

In the present analysis, we accurately simulated the US general population structure, vaccination status and prevalence, and the ILI incidence pattern of the studied flu season. The incidence data represented only the proportion of PCP visits due to ILIs, which is known to be relatively low (Biggerstaff et al., 2014; Peppa et al., 2017) and likely lead to an underestimation.

One of the base case scenarios only considered the effects of probiotics among the unvaccinated individuals, in line with the results of the Cochrane meta-analysis. The results of the model were therefore conservative, since probiotics can potentially have effects on the vaccinated individuals (Yaqoob, 2014; Zimmermann and Curtis, 2018). Another aspect of probiotic intake not captured in the model concerns the role probiotics may play in the reduction of antibiotic use (King et al., 2018). The CDC estimates that of the 44% of outpatient antibiotics prescribed to treat patients with respiratory conditions, half are unneeded (CDC Newsroom, 2016). Reduced antibiotic use and the associated risk of antibiotic resistance may have considerable public health relevance as well as additional cost consequences (Michaelidis et al., 2016).

A limitation of our analysis is that probiotics were considered as a general category and not included in our assessment at the strain level. Different probiotic strains may have different effects on RTIs, but because our evaluation was based on two specific meta-analyses, we effectively included all relevant probiotic strains evaluated in one or more of the clinical trials that were pooled via these meta-analyses. Further, data available for probiotic use in the USA are not segregated based on specific strains. Advances have been made in unraveling the wide array of molecular mechanisms by which probiotic organisms can interact with host cells and on understanding how this might translate into a clinical effect (Kleerebezem et al., 2019). Certain health benefits depend on core properties that are conserved among different probiotics, while other benefits appear to be more strain specific (Hill et al., 2014; Sanders et al., 2018). An expression of shared efficacy among many different strains may derive from common mechanisms among taxonomic groups that are at a higher order than a strain, such as species or genus. An example of such a shared mechanism is production of short-chain fatty acids. In the case of prevention of RTIs, the mechanism is not known, although it may likely involve probiotic interactions with the immune system. For purposes of this analysis, we consider it sufficient to note that studies included in the meta-analyses, upon which it is based, included interventions from a variety of different *Lactobacillus* and *Bifidobacterium* strains.

As in the previous French and Canadian analyses (Lenoir-Wijnkoop et al., 2015, Lenoir-Wijnkoop et al., 2016), the cost of probiotics was not included as a factor in our model. Although costs of probiotic foods and dietary supplements do not weigh on the health care payers’ resources, their purchase may put an extra burden on the average household expenses. Due to an absence of reliable information and a great variation in products with a wide range of unit prices, it was not possible to evaluate how much this would represent. However, these additional household costs would probably be offset by other expenses, such as costs related to self-medication and purchase of over-the-counter drugs (Klepser, 2014) and costs related to informal care for sick children or the elderly (Chari et al., 2015) and associated with missed schooldays (Li and Leader, 2007; NCCID, 2014).

## Conclusion

The model demonstrated a positive impact of probiotic consumption on health outcomes in flu-like RTI and the associated patient burden by reducing the number of RTI episodes, the number of days patients spent with RTI symptoms, and the need for antibiotics. Improved patient outcomes translated into considerable cost savings for both the payer and society. These results suggest that recommending daily probiotic consumption may be justified for particular at-risk populations, such as children or individuals with a shared indoor environment, for which this study shows a higher incremental benefit. Such action should be in combination with a cost-effectiveness analysis of implementation to further define the extent to which probiotics contribute to reducing both health care spending and out-of-pocket costs for the management of flu-like infections.

## Data Availability

All sources for the data used in this study are mentioned and referenced in the manuscript. Detailed calculations of the model outcomes are available upon request directed to Dr. Christa Broholm (DKCHBR@chr-hansen.com).

## Author Contributions

IL-W initiated and coordinated the study; DK and IL-W contributed to the conception and design of the study; DK developed the model, organized the database, and performed the statistical analyses; MS, DM, and DT validated country-specific assumptions; IL-W wrote the first draft of the manuscript; MS, DT, and CB wrote sections of the manuscript; DK and CB adapted the tables and figures; and all authors contributed to the manuscript revision and read and approved the submitted version.

## Funding

This project was supported by an unrestricted grant from Chr. Hansen. The funder was not involved in the study design, collection, analysis, interpretation of data or the decision to submit it for publication.

## Conflict of Interest Statement

Author CB is an employee at Chr. Hansen. Author DK is employed by IQVIA. Author MS serves as an executive science officer for the International Scientific Association for Probiotics and Prebiotics. She also reports personal fees outside the submitted work from the following entities: International Scientific Association for Probiotics and Prebiotics, Pharmavite, CD Investments, Dannon, Danone USA, Yakult, California Dairy Research Foundation, Winclove BioSciences BV, Nestle, Williams Mullen, New Chapter, Dutch Mill, Clorox, Pfizer, Visalia Dairy Company, Procter & Gamble, Kelley Drye & Warren LLP, Kellogg, Trouw Nutrition, Kerry, JHeimbach LLC, General Mills, Probi, and Medscape. Author DM declares consulting for Bayer and Pharmavite.

The remaining authors declare that the research was conducted in the absence of any commercial or financial relationships that could be construed as a potential conflict of interest.
