# Corrigendum: Probiotics Reduce Health Care Cost and Societal Impact of Flu-Like Respiratory Tract Infections in the USA: An Economic Modeling Study

**DOI:** 10.3389/fphar.2019.01182

**Published:** 2019-10-11

**Authors:** Irene Lenoir-Wijnkoop, Dan Merenstein, Daria Korchagina, Christa Broholm, Mary Ellen Sanders, Dan Tancredi

**Affiliations:** ^1^Department of Pharmaceutical Sciences, University of Utrecht, Utrecht, Netherlands; ^2^Family Medicine Department, Georgetown University, Washington, DC, United States; ^3^Real World Insights, IQVIA, Paris, France; ^4^Chr. Hansen A/S, Human Health Innovation, Hoersholm, Denmark; ^5^Dairy & Food Culture Technologies, Centennial, CO, United States; ^6^Department of Pediatrics and the Center for Healthcare Policy and Research, University of California, Davis, Sacramento, CA, United States

**Keywords:** probiotics, health economics, respiratory tract infection, influenza, cost savings

In the original article, there was an error. There is a mistake in the **Funding** statement. The mention that the funder, Chr. Hansen, was not involved in the writing of the manuscript is incorrect. A correction has been made to the **Funding** statement:

“This project was supported by an unrestricted grant from Chr. Hansen. The funder was not involved in the study design, collection, analysis, interpretation of data or the decision to submit it for publication”

Furthermore, there is a mistake in the **Conflict of Interest Statement**. The declaration that the author DM consulted Bayer and Pharmative was erroneously omitted. A correction has been made to the **Conflict of Interest Statement**:

“Author CB is an employee at Chr. Hansen. Author DK is employed by IQVIA. Author MS serves as an executive science officer for the International Scientific Association for Probiotics and Prebiotics. She also reports personal fees outside the submitted work from the following entities: International Scientific Association for Probiotics and Prebiotics, Pharmavite, CD Investments, Dannon, Danone USA, Yakult, California Dairy Research Foundation, Winclove BioSciences BV, Nestle, Williams Mullen, New Chapter, Dutch Mill, Clorox, Pfizer, Visalia Dairy Company, Procter & Gamble, Kelley Drye & Warren LLP, Kellogg, Trouw Nutrition, Kerry, JHeimbach LLC, General Mills, Probi, and Medscape. Author DM declares consulting for Bayer and Pharmavite. The remaining authors declare that the research was conducted in the absence of any commercial or financial relationships that could be construed as a potential conflict of interest.”

Lastly, there was a mistake in the **Methods**, section **Model Inputs and Data Sources**, sub-section **Probiotic Effect**. The RTI incidence and antibiotic prescription rate was provided in the incorrect unit. A correction has been made to the **Methods**, section **Model Inputs and Data Sources**, sub-section **Probiotic Effect**, paragraph one:

“The clinical effects of probiotics were obtained from the meta-analyses published by the YHEC (King et al., 2014) and Cochrane (Hao et al., 2015). These were used to conduct two independent scenario analyses comparing generalized probiotic use versus non-use, each based on different assumptions: YHEC showed a significantly shorter duration of −0.77 days [−1.50 to −0.04] on an average duration of 7.4 days per episode of RTI, among otherwise healthy children and adults taking probiotics compared to those taking placebo. The Cochrane study reported that probiotics significantly reduced RTI duration by 1.89 days [1.75 to 2.03] per episode of an average duration of 8.82 days and RTI incidence by 30% (RR = 0.70 [0.50 to 0.84]) (Table 1). The authors also found a significant reduction of the antibiotic prescription rate of 35% (RR = 0.65 [0.45 to 0.94]), which was applied to both scenarios.” Additionally, the YHEC meta-analysis studied the impact of probiotics on work absenteeism. The reported standardized mean difference (SMD) in the number of days absent from work was used to estimate the impact of probiotics on productivity loss. The Cochrane meta-analysis focused on unvaccinated individuals; therefore, no impact of probiotics sourced from the Cochrane meta-analysis was applied in vaccinated patients.

The authors apologize for these errors and state that they do not change the scientific conclusions of the article in any way.

